# Ultra-long Pt nanolawns supported on TiO_2_-coated carbon fibers as 3D hybrid catalyst for methanol oxidation

**DOI:** 10.1186/1556-276X-7-237

**Published:** 2012-06-26

**Authors:** Yu-Lin Shen, Shih-Yun Chen, Jenn-Ming Song, In-Gann Chen

**Affiliations:** 1 Graduate Institute of Applied Science and Technology, National Taiwan University of Science and Technology, Taipei, 106, Taiwan; 2 Department of Materials Science and Engineering, National Taiwan University of Science and Technology, Taipei, 106, Taiwan; 3 Department of Materials Science and Engineering, National Chung Hsing University, Taichung, 402, Taiwan; 4 Department of Materials Science and Engineering, National Cheng Kung University, Tainan, 701, Taiwan

**Keywords:** Pt nanowires, Hybrid catalyst, Methanol oxidation, Thermally activated photoreduction

## Abstract

In this study, TiO_2_ thin film photocatalyst on carbon fibers was used to synthesize ultra-long single crystalline Pt nanowires via a simple photoreduction route (thermally activated photoreduction). It also acted as a co-catalytic material with Pt. Taking advantage of the high-aspect ratio of the Pt nanostructure as well as the excellent catalytic activity of TiO_2_, this hybrid structure has the great potential as the active anode in direct methanol fuel cells. The electrochemical results indicate that TiO_2_ is capable of transforming CO-like poisoning species on the Pt surface during methanol oxidation and contributes to a high CO tolerance of this Pt nanowire/TiO_2_ hybrid structure.

## Background

In recent years, direct methanol fuel cell (DMFC) has attracted great attention as an alternative power source because of their many advantages, including light weight, high power density, portability, and storage of liquid fuel [1,2]. For most of the common DMFC devices, Pt-based catalysts have been used as an anode because of their outstanding performance in catalyzing the dehydrogenation of methanol. However, the commercialization of DMFC device still faces some problems, such as CO adsorption on Pt catalysts and thus poisoning [3,4]. Even a low concentration of CO will cause a remarkable decrease in catalytic activity of Pt-based electrodes. Therefore, the majority of the Pt-based electrocatalyst research has focused on this topic.

In addition to alloying [5,6], there are two other strategies for improving the Pt-based catalyst's performance. The first is to modify the morphology of Pt nanostructure, including the shape as well as the dimension. It has been demonstrated that changing the morphology of the Pt nanostructure from nanoparticle (NP) to nanowire (NW) can enhance the electrocatalytic activity of the catalysts, due to the large side surface which is able to provide additional catalytic active facets [7,8]. A great deal of effort has been devoted to the synthesis of one-dimensional Pt nanostructures; however, it still remains a huge challenge to synthesize long and oriented single-crystalline Pt NWs without temperates and surfactants. Lee et al. [9,10] have demonstrated the synthesis of single crystalline Pt NWs on polymeric, ceramic, or metallic substrate by a polyol process, combined with a trace addition of iron species (Fe^2+^ or Fe^3+^) and poly(vinylpyrrolidone) (PVP) as the surfactant. Cetyltrimethylammonium bromide (CTAB) has also been applied in the reduction of Pt ions to Pt NWs [7,11]. Moreover, without using templates and surfactants, HCOOH [12-15] and vitamin B_2_[16] have been suggested respectively to act as reductant agents in the chemical routes for the synthesis of Pt NWs. Through the above processes, the Pt NWs produced are extremely fine (mostly less than 10 nm in diameter) but exhibit a limit in length of about 200 nm so that their aspect ratios do not exceed 50.

The next tactic to enhance the catalyst activity and CO-tolerance is the development of new composites and catalytic supporting materials. In the last decade, the addition of oxides has been generally accepted as an efficient way to improve catalytic activity of platinum and its CO-tolerance for methanol electro-oxidation. For example, Cui et al. [17] found that the Pt-WO_3_/C composite catalysts exhibit excellent catalytic activity and stability for methanol electro-oxidation because WO_3_ is able to form the hydrogen-tungsten-bronze compound, which facilitates dehydrogenation during methanol oxidation reaction. RuO_2_, ZrO_2_, and MgO have also been studied and it was found that they can improve the catalytic performance of Pt [18-20]. A promising breakthrough is that the composites of Pt or Pt-Ru nanoparticles and semiconductor catalysts such as TiO_2_ and CeO_2_ have been developed as the anode for oxidation of methanol or ethanol [21-24]. It has been proposed that the Pt-CeO_2_ composite catalyst has a higher activity than Pt catalyst because CeO_2_ makes CO electro-oxidation easier. The redox reaction on the surface of CeO_2_ mixing with Pt particles causes the oxidation of CO to CO_2_ and thus gives rise to a better performance. Drew et al. [22] verified that TiO_2_ can enhance the current generation especially under ultraviolet (UV) light irradiation during the electrochemical testing and suggested that the holes formed upon UV illumination are consumed in methanol oxidation and bring about the additional current.

In view of the above description, this study aims to develop highly efficient three-dimensional composite electrode catalysts with Pt NWs grown on oxide-coated carbon fibers. A recently developed process, thermally assisted photoreduction (TAP) [25,26], was applied to prepare ultra-long metallic nanowires through the photocatalysis of TiO_2_. According to the Honda-Fujishima effect [27], electrons and holes on the surface of TiO_2_ films can be activated by UV light, which enables the reduction of metallic ions from the solution. The photoreduction process of metallic ions (M^*+*^) can be expressed briefly as follows,

(1) $$TiO_{2}\overset{h\nu }{\to }h^{+}+e^{-}$$

(2) $$M^{+}+e^{-}\overset{}{\to }M^{o}$$

Based on this, metallic nanowires can be formed on the surface of thin-film TiO_2_ via the photoreduction of metallic ions under certain irradiating and heating conditions. Instead of commonly used H_2_PtCl_6_, Na_2_Pt(OH)_6_ was selected as the precursor in this work.

## Methods

**Figure 1:**
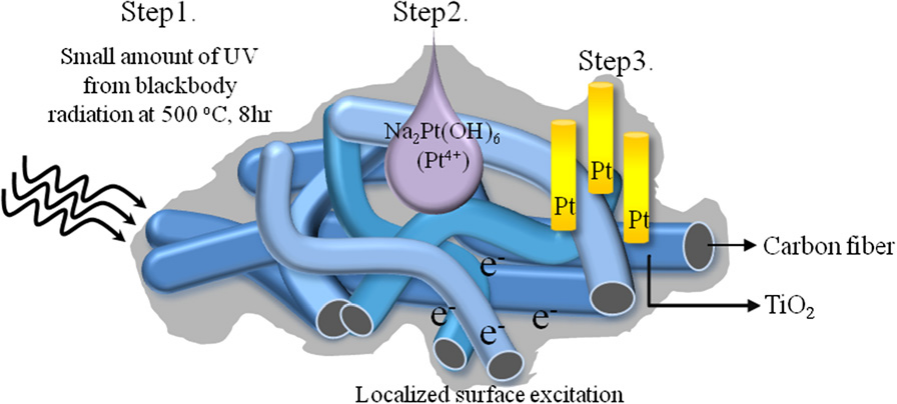
Schematic illustration of the main steps for forming Pt nanowires by thermally assisted photoreduction.

The whole route for the synthesis of Pt NWs was illustrated in Figure 1. To make gel coating TiO_2_ films, the carbon fibers sheet in the size of 1 × 1 cm was dipped into the TiO_2_ solution. The carbon fiber sheets used were with a thickness of 360 μm and basis weight of 125 g/m^2^. After dipping, the weight gain per sample by absorption of TiO_2_ solution was 0.035 g. The dipped samples were then annealed at 500°C for 8 h in an oxygen atmosphere to obtain well crystallized anatase TiO_2_ (step 1 in Figure 1). The TiO_2_ solution used was prepared with isopropylalcohol (IPA)/titanium isopropoxide (TTIP)/hydrogen chloride (HCl) with the volume ratio 170:12:0.4 and stirred for 10 min at room temperature (20°C) before aging for 2 days. The weight of TiO_2_ on a 1 cm^2^ carbon fiber sample after annealing was measured to be 0.012 g (3.1 × 10^−3^ cm^3^ in volume). Fifteen microliters of 0.05 M Na_2_Pt(OH)_6_ aqueous salt solution was dropped on the TiO_2_ coated substrates (step 2). Afterward, the samples were isothermally heated at 300°C for 3 h in air by an infrared furnace, followed by a furnace-cooling to the ambient temperature (namely, the post thermal treatment, step 3 in Figure 1).

It has been demonstrated that the extent of UV exposure on the TiO_2_ film affects the degree of excitation and thus strongly influences the shape and dimension of the reduced metallic structure [25]. In order to obtain reduced Pt with different morphologies and sizes, we varied the conditions of pre-UV exposure treatment performed on the annealed TiO_2_-coated carbon cloths before the growth of the nanowires (between the step 1 and step 2). The conditions included (1) no pre-UV light exposure, (2) pre-UV exposure on only one side of the carbon cloths for 48 h, and (3) pre-UV exposure on each side of carbon cloths for 12 h alternately to reach a total exposure time of 48 h. It is assumed that on the exposed side of the carbon cloth the TiO_2_ was excited. Therefore, the whole TiO_2_ film was presumed to be fully excited.

Electrochemical measurements of the hybrid electrocatalysts were performed in a three-electrode cell using an Autolab PG302N work station (Metrohm, Autolab BV, Utrecht, The Netherlands) at room temperature to evaluate their catalytic performance. Carbon cloths (0.25 cm^2^) with Pt-TiO_2_ catalysts were the working electrode. A Pt rod and Ag/AgCl were used as counter and reference electrodes, respectively. A solution of 1 M CH_3_OH and 0.5 M H_2_SO_4_ was used as the electrolyte. All the reagents used were of analytical grade. The cyclic voltammetry data for methanol electro-oxidation were recorded in the potential range of −0.2 to 1.0 V vs. Ag/AgCl with a scan rate of 20 mVs^−1^.

## Results and discussion

**Figure 2:**
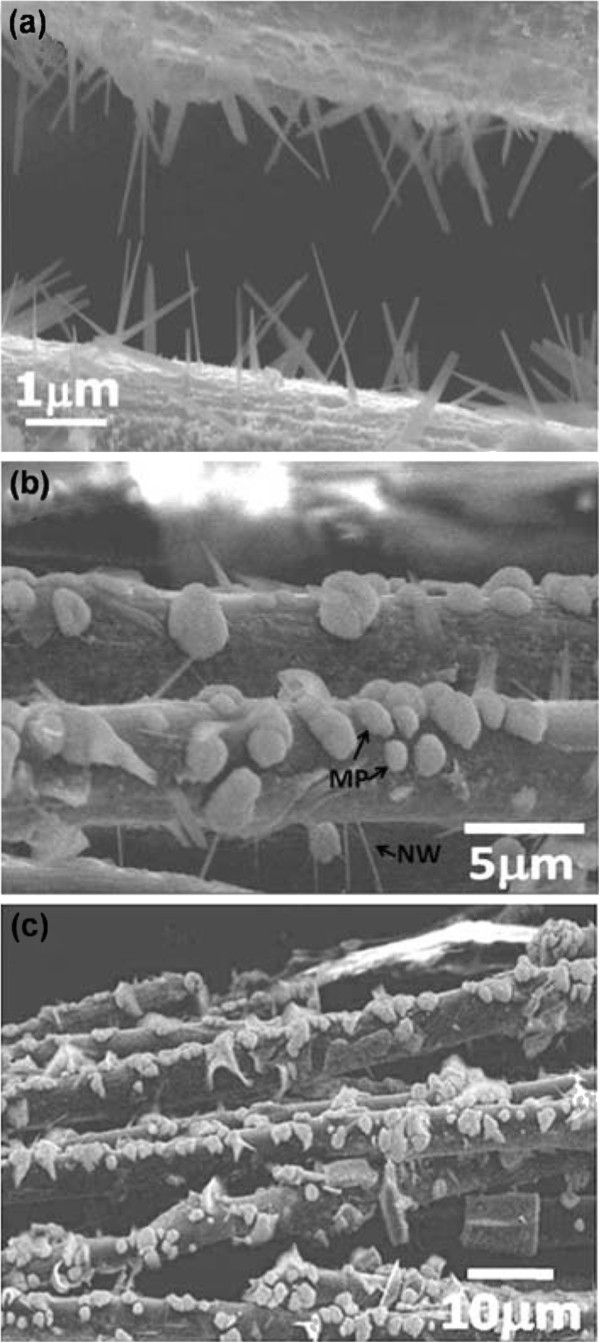
**Morphologies of the reduced Pt on TiO**_**2**_**coated carbon cloths with different UV exposure conditions.** (**a**) no pre-UV-irradiated (**b**) one side-UV irradiated, (**c**) both side-UV irradiated.

After heating Na_2_Pt(OH)_6_ solution at 300°C for 3 h, the scanning electron microscopy image shown in Figure 2a illustrates that all the Pt salt was transformed into Pt NWs grown radially on both sides of the carbon cloths under no pre-UV exposure condition (condition 1), with an average diameter of about 40 nm and remarkable length of 1 to approximately 2 μm. Most of the NWs exhibit an aspect ratio ranging from 25 to 50. Of particular interest is, after long duration of pre-UV irradiation, microsized Pt nodules formed on the UV-exposed portion of the carbon cloths instead of nanowires. Figure 2b indicates that the samples under the condition 2 showed a Janus feature. That is, Pt microparticles (MP) grew on the exposed side (the upper side) of the carbon cloth, while the unexposed side (the underneath side) was with Pt nanowires. Furthermore, the surface of the two-side exposed carbon cloths in the condition 3 was completely covered with Pt microparticles (Figure 2c). Accordingly, these three structures are designated as NW/TiO_2_, NW + MP/TiO_2_, and MP/TiO_2_, respectively.

**Figure 3:**
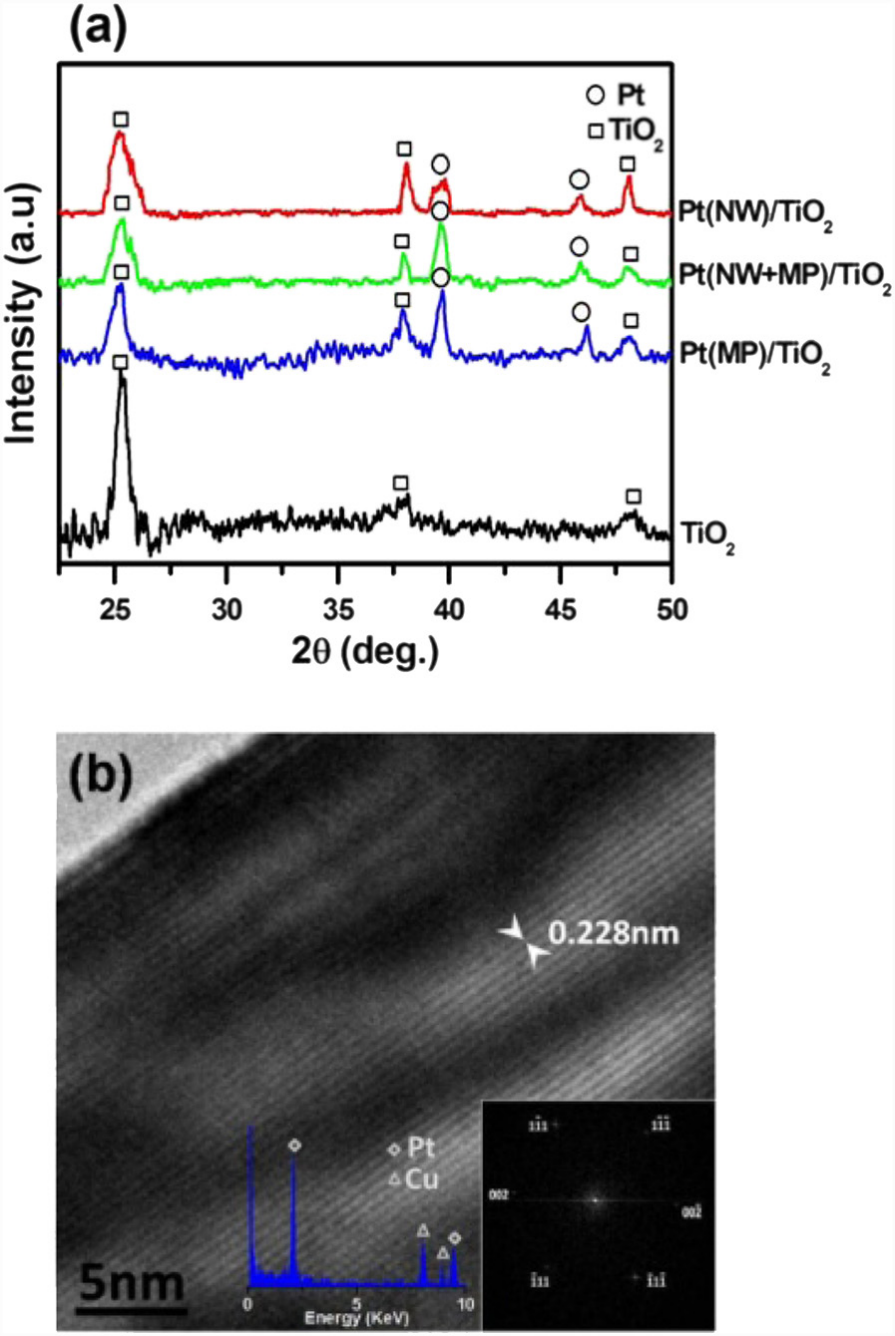
**XRD patterns of Pt/TiO**_**2**_**hybrid structures, the HR-TEM image and EDS analytical results of a Pt nanowire.** (**a**) XRD patterns of Pt/TiO_2_ hybrid structures shown in Figure 2, (**b**) the HR-TEM image and EDS analysis of a Pt nanowire (the Cu signal comes from the Cu grids).

The grazing angle X-ray diffraction (XRD) patterns, in Figure 3a, verify that those reduced structures on the TiO_2_ coating were all platinum. Figure 3b shows a high-resolution transmission electron microscopy (HR-TEM) image and energy dispersive spectrometer (EDS) spectrum of a Pt nanowire. The HR-TEM image shows the lattice spacing between the {111} planes to be 0.228 nm, which was in agreement with the value in the bulk Pt crystal, suggesting the Pt NWs grew along <111> axes, and the EDS spectrum demonstrates that the NWs thus produced were pure Pt without any detectable impurity. The inserted electron diffraction pattern constructed by the fast Fourier transform also verifies the preferred growth direction to be <111>. Similar results have been reported in our previous work [28].

**Figure 4:**
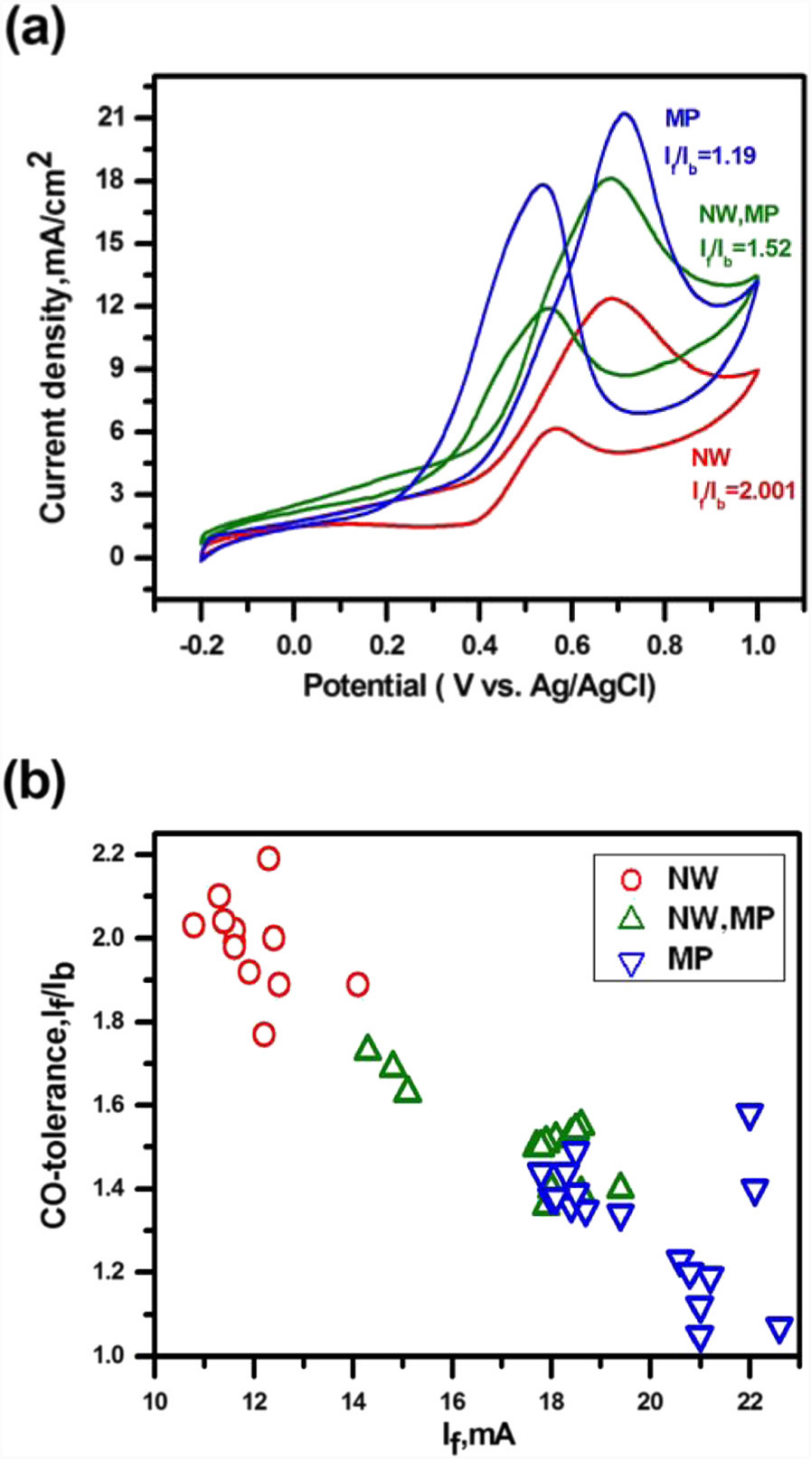
**Electrochemical performance of the Pt/TiO**_**2**_**hybrid structures with different morphologies.** Electrochemical performance of the Pt/TiO_2_ hybrid structures with different morphologies in the solution of 1 M CH_3_OH and 0.5 M H_2_SO_4_ with a scan rate of 20 mVs^−1^: (**a**) cyclic voltammograms and (**b**) CO tolerance vs. forward peak current density.

Figure 4a illustrates the standard cyclic voltammogram curves (the 5th cycle) for methanol electro-oxidation on carbon fiber-supported Pt-TiO_2_ structures. For all the samples, a methanol oxidation peak appeared in the forward scan and a reoxidation peak was detected on the reverse sweep. Obtained from the cyclic voltammetry (CV) curves in Figure 4a, the maximum current density of the forward peak (*I*_f_) and that of the backward peak (*I*_b_), as well as their ratio, *I*_f_/*I*_b_, are given in Table 1, indicating the differences in electrochemical behavior. The forward and backward peak current densities (*I*_f_ and *I*_b_) of the MP/TiO_2_ samples were greater than NW + MP/TiO_2_, which was in turn higher than NW/TiO_2_ samples. Also, the onset potential in the forward scan of the MP/TiO_2_ samples was 0.349, slightly lower than the others. Remarkably, the *I*_f_/*I*_b_ value, which could be used to infer the CO tolerance of the catalysts, in a decreasing order was NW/TiO_2_ (2.00), NW + MP/TiO_2_ (1.52), and then MP/TiO_2_ (1.19). According to the results of Figure 4a, Figure 4b combines the data distribution of the forward peak current density and corresponding CO tolerance, indicating that NW/TiO_2_ catalysts exhibited higher CO tolerance but inferior current generation ability compared to MP/TiO_2_ mainly due to the limited amount of reduced Pt.

**Table 1:** **Electrochemical characteristics of the specimens during CV analysis (derived from the CV curves in Figure**4**a)**

**Catalysts**	**Pt NW/TiO**_**2**_	**Pt NW + MP/TiO**_**2**_	**Pt MP/TiO**_**2**_
*I*_f_ current density (mA)	12.37	18.14	21.21
*I*_b_ current density (mA)	6.18	11.93	17.82
*I*_f_/*I*_b_	2.00	1.52	1.19
Onset potential (V)	0.385	0.383	0.349

The excellent performance in CO tolerance of the NW/TiO_2_ samples can be explained that the high aspect ratio of the NWs leaving most of the TiO_2_ film uncovered and allowing for subsequent reactions [22], which were similar to those in the CeO_2_-Pt composites [29].

(3) $${\text{TiO}}_{2}+{\text{H}}_{2}\text{O}\to {\text{TiO}}_{2}-{\text{OH}}_{\text{ads}}+{\text{H}}^{+}+{\text{ e}}^{-}$$

(4) $$\text{Pt}-{\text{CO}}_{\text{ads}}+{\text{TiO}}_{2}-{\text{OH}}_{\text{ads}}\to \text{Pt}+{\text{TiO}}_{2}+{\text{CO}}_{2}+{\text{H}}^{+}+{\text{ e}}^{-}$$

The OH_ads_ species (ads, adsorbed) on TiO_2_, obtained through the activation of water, can transform CO-like poisoning species (CO_ads_) on the Pt surface, produced by the methanol dehydrogenation, to CO_2_ and release active Pt for further catalysis.

The TAP process needs to be improved to obtain a higher population of Pt NWs and thus raises the catalytic activity for methanol oxidation. If this is done, it can be expected that the excellent performance in CO tolerance can be maintained because the high-aspect-ratio Pt NWs occupy only a small part of the fiber surface and leave most the TiO_2_ film free for oxidation of CO.

## Conclusions

By means of templateless and surfactant-free method, TAP, this study successfully prepared carbon fibers supported Pt nanowires/TiO_2_ composite electrocatalysts, which show great potential for use as active anode in direct methanol fuel cells. Analytical results suggest that the Pt nanowires were single crystalline with a preferred <111> growth direction and exhibited an aspect ratio ranged between 25 and 50. This Pt nanowire/TiO_2_ hybrid structure possessed high CO tolerance because TiO_2_ enhances CO electro-oxidation and thus increases CO poisoning resistance. An optimal performance in catalytic activity for methanol oxidation and CO tolerance can be expected if the density of the Pt nanowires is increased further.

## Competing interests

The authors declare that they have no competing interests.

## Authors’ contributions

Y-LS carried out the main part of synthetic and analytical works, participated in the sequence alignment and drafted the manuscript. S-YC participated in the discussion of the growth mechanism. J-MS participated in the design of the study, draft preparation and coordination. I-GC conceived of the study and participated in its design. All authors read and approved the final manuscript.

## Authors’ information

YLS is a PHD student at Graduate Institute of Applied Science and Technology, National Taiwan University of Science and Technology, Taipei 106, Taiwan. SYC is an associate professor in the Department of Materials Science and Engineering, National Taiwan University of Science and Technology, Taipei 106, Taiwan. JMS is an associate professor in the Department of Materials Science and Engineering, National Chung Hsing University, Taichung 402, Taiwan. IGC is a distinguished professor from the Department of Materials Science and Engineering, National Cheng Kung University, Tainan 701, Taiwan.
